# Shared decision-making and the caregiver experience in tuberous sclerosis complex: results from a UK survey

**DOI:** 10.1186/s13023-023-02677-7

**Published:** 2023-04-11

**Authors:** Hanna Skrobanski, Kishan Vyas, Sally Bowditch, Lena Hubig, Edward Dziadulewicz, Louise Fish, Pooja Takhar, Siu Hing Lo

**Affiliations:** 1 Acaster Lloyd Consulting Ltd, London, UK; 2 Jazz Pharmaceuticals, Inc., London, UK; 3 Tuberous Sclerosis Association, London, UK; 4 Genetic Alliance UK, London, UK

**Keywords:** Shared decision-making, COVID-19, Tuberous sclerosis complex, Caregiver burden

## Abstract

**Background:**

Tuberous sclerosis complex (TSC) is a rare genetic condition commonly accompanied by neurological and neuropsychological disorders, resulting in a high burden of illness for individuals and a substantial impact on their caregivers. Due to the diversity and complexity of clinical manifestations, patients with TSC need aligned multidisciplinary healthcare services starting in childhood through to adulthood. However, patients and caregivers are sometimes dissatisfied with the care provided, for which one of the most common reasons is a lack of involvement in clinical decision-making. Shared decision-making, whereby clinicians make clinical management decisions together with patients and their caregivers, is advocated for in the management of epilepsy, but evidence of its benefit in managing TSC is currently lacking. In this cross-sectional, UK-based analysis we used an online survey to capture the experiences of primary caregivers for individuals with TSC, including the impact on work productivity, clinical shared decision-making, satisfaction with care, and the impact of the coronavirus disease 2019 (COVID-19) pandemic.

**Results:**

In total, 73 eligible caregivers provided consent (analysis set), with 14 completing the survey partially and 59 completing the full survey. Many caregivers (72%) reported receiving recommendations about new treatments from their doctor and discussing the treatment together, with a high proportion (89%) preferring that treatment was initiated at a low dose. Most caregivers (69%) were satisfied or extremely satisfied with pediatric TSC healthcare services, but only 25% were satisfied or extremely satisfied with the transition to adult TSC healthcare services. Several (n = 30) caregivers specified the impact of caring on their work productivity and career in optional open-ended survey responses. Finally, 80% of caregivers indicated that the COVID-19 pandemic had a “large” or “very large” impact on their caring activities, negatively affecting the emotional wellbeing and behavior of individuals with TSC, and caregivers’ ability to work and arrange medical appointments.

**Conclusions:**

Caregivers largely feel involved in treatment decisions, and the majority were satisfied with healthcare services for children with TSC. However, many highlighted the need for an improved transition from pediatric to adult healthcare services. The survey also showed that COVID-19 has considerably affected caregivers and individuals with TSC.

**Supplementary Information:**

The online version contains supplementary material available at 10.1186/s13023-023-02677-7.

## Background

Tuberous sclerosis complex (TSC) is a rare genetic condition characterized by multiple benign tumors most commonly affecting the brain, skin, lungs, kidneys, eyes, and heart [1]. Although the clinical manifestations of TSC can vary among individuals, neurological and psychological disorders are also commonly associated with the condition, [1] which can result in a high burden of illness for individuals [1] and a need for lifetime medical management [2].

Caring for individuals with TSC can have a considerable impact on families and households, [3–5] with caregivers of individuals with TSC often experiencing a lower health-related quality of life (HRQL) and more symptoms of depression than non-caregivers [6].

Given the continual medical management needs of individuals with TSC, the diverse and complex clinical presentation of TSC, and the impact on caregivers and families, there is a considerable demand for multidisciplinary healthcare services from childhood through to adulthood. Aligning these factors can make management challenging, and previous studies have found that individuals with TSC and their caregivers are not always satisfied with the care provided [7]. For example, in a survey with 143 individuals with TSC who participated in the TuberOus Sclerosis registry to increase disease awareness (TOSCA) study, ~ 20% were dissatisfied with their overall care, and up to ~ 50% did not have clarity whether their treatment adhered to clinical guidelines [7]. The same survey also identified the transition from pediatric to adult care as an area of concern, with only 37% of adult patients with TSC reporting the transition process as smooth [7].

A common reason for dissatisfaction with patient care is a lack of involvement in clinical decision-making [8]. Shared decision-making is an approach where clinicians and patients (and their caregivers) make clinical management decisions together using the best available evidence [8]. There is no current evidence looking specifically at the experience of shared decision-making in the management of individuals with TSC, although the benefits of such an approach have been advocated for in the management of epilepsy, [8] one of the neurological manifestations of TSC [1]. Similarly, another area in which there are no published studies is the impact of the coronavirus disease 2019 (COVID-19) pandemic on individuals with TSC and their caregivers.

A UK survey was conducted from May to July 2021 to examine the burden of TSC for primary caregivers and their families [9]. From a cohort of 73 participants, primary caregivers (n = 69) spent a mean (standard deviation; SD) of 104.3 (51.7) hours caring in the previous week, reporting higher rates of anxiety and depression than UK population norms and a considerable family burden. Increased seizure frequency (> 12 seizures vs. 0 seizures in the previous week) was associated with increased hours spent caring by primary caregivers (*p* = 0.01), decreased family functioning (*p* = 0.03), and decreased parent HRQL (*p* = 0.03).

Although the present study is based on the same survey of caregivers of individuals with TSC in the UK, [9] here we expanded on those findings and explored additional, broader aspects of the caregiver experience. For example, we aimed to determine the impact of caring on the participants’ work and careers and their experience of shared decision-making in relation to the treatment of seizures, as well as their experiences of and satisfaction with healthcare services in the UK and, where applicable, the transition from pediatric to adult care. This survey also aimed to capture caregivers’ feedback on the impact of the COVID-19 pandemic on both their dependent with TSC and their caregiving responsibilities.

## Methods

A cross-sectional online survey of caregivers of individuals with TSC in the UK, recruited via the Tuberous Sclerosis Association (TSA), was conducted between May and July 2021. Methodological details of the survey have been published recently [9]. In brief, eligible participants were unpaid primary caregivers of an individual with TSC, living with the individual with TSC that they care for, aged ≥ 18 years, and a UK resident. The target sample size was 100 caregivers of individuals with TSC, which was based on an informal recruitment feasibility assessment by the TSA. Individuals completed a screening survey to ensure eligibility; those eligible were provided with additional survey details and asked to complete a consent form ahead of progressing to the main survey. A £20 donation was made by GW Pharmaceuticals, now part of Jazz Pharmaceuticals, Inc., to the TSA for every survey recruit.

The survey was developed following a literature review [9] and feedback from the TSA. This survey included both closed- and open-ended questions that were specifically developed to capture different aspects of caring for an individual with TSC. For the purposes of this publication, the survey included questions on the following topics:

- Individuals with TSC, caregiver, and household characteristics, assessed through multiple-choice questions and numerical input.
- Financial and social support received by individuals with TSC and their household, assessed through multiple-choice questions.
- The impact of caring on the primary caregiver’s work and career, assessed through an open-ended question allowing for free-text comments.
- Caregiver experience of shared decision-making in relation to antiseizure medications (ASMs) taken by the individual they care for and their preference for treatment dose when they start taking a new ASM, assessed through multiple-choice questions.
- Caregiver satisfaction with pediatric and/or adult healthcare services used by the individual with TSC and their experience of transition from pediatric to adult healthcare services (if applicable), assessed through multiple choice questions; an additional open-ended question allowed for free-text comments.
- The impact of the COVID-19 pandemic on the caregiver’s caring responsibilities, assessed through multiple-choice questions; an additional open-ended question allowed for free-text comments.

Participants had to answer “required” questions and were able to return to previous questions to review answers. It was not possible to prevent multiple entries from a single participant due to anonymity requirements.

All survey responses were included in the analysis, and no missing data were imputed. Respondents’ data were only included in individual analyses where it was complete for all included outcomes. Closed-ended responses were summarized using descriptive statistics, including count and percent data for categorical variables, and mean and SD for continuous variables. All analyses were conducted in R 4.1.0 (R Foundation, Vienna, Austria) [10]. Qualitative content analysis was conducted with Microsoft Excel for open-ended responses.

## Results

### Individuals with TSC, Caregiver, and Household characteristics

**Fig. 1:**
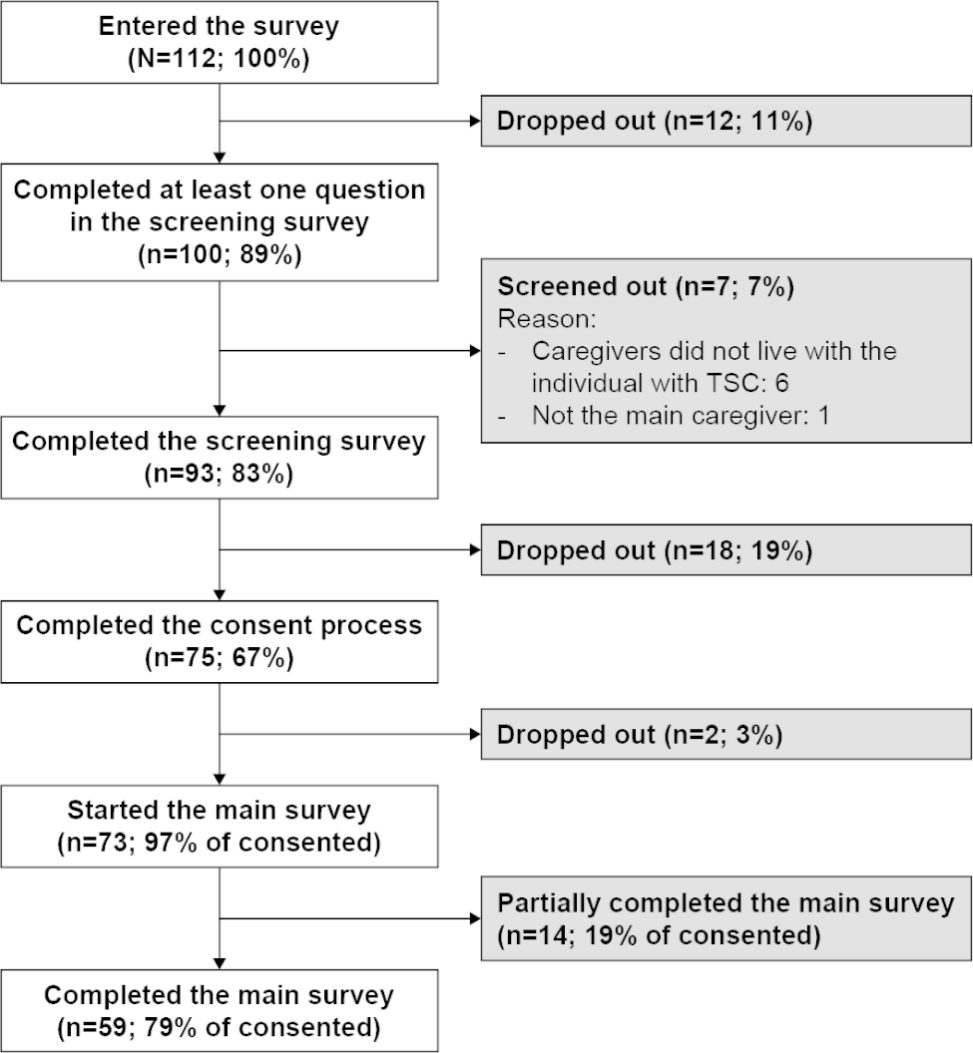
Participant flow from enrolment to completion for the online survey of primary caregivers of individuals with TSC Abbreviations: TSC = tuberous sclerosis complex

The number of participants taking part in each stage of the study, including the screening survey, consent, and main survey, has been published previously [9] and is shown in Fig. 1. Briefly, 73 caregivers were deemed eligible and provided consent (analysis set); of these, 14 caregivers completed the survey partially and 59 completed the full survey.

Individuals with TSC, caregiver, and household characteristics have been described in detail in a previous publication [9]. In brief, the majority (90%) of caregiver participants were parents of their dependent with TSC and most were female (95%), either employed part time (38%) or staying at home full time (33%). 47% were caregivers of children or adolescents under the age of 18 years (mean [SD] age of individuals with TSC: 20.0 [13.5] years). On average (mean [SD]), 95% of individuals had experienced an epileptic seizure of any type during their lifetime, of whom 94% were currently taking treatment for seizures.

### Financial and Social Support received by individuals with TSC and their Household, and Use of National Health Service (NHS) by Household

Details of the financial and social support received are shown in Table 1. The majority of individuals (82%) received disability allowance for their condition, with just under a third receiving support from social services.

**Table 1:** Financial and social support received by individuals with TSC and their household

Characteristics				
**Financial and social support received by individual with TSC (n = 71)**	Disability allowance		58 (82)	
	Support from social services		21 (30)	
	Support from social worker		13 (18)	
	Help completing benefit applications		10 (14)	
	Psychological counseling		5 (7)	
	None		9 (13)	
**Financial support received by primary caregiver (n = 64)**	Carer allowance		23 (36)	
	Carer credit		2 (3)	
	Other		3 (5)	
	None		36 (56)	
		**Primary caregiver ** **(n = 64)**	**Partner/spouse ** **(n = 43)** ^**a**^	**Other household members (n = 15)** ^**b**^
**Use of NHS services by household (n = 64)**	Yes	24 (38)	6 (14)	1 (7)
	No	40 (63)	34 (79)	13 (87)
	Prefer not to answer	0	3 (7)	1 (7)
	General practice^c^	19 (79)	6 (100)	1 (100)
	Counseling^c^	12 (50)	2 (33)	0
	Respite care^c^	7 (29)	1 (17)	0
	Hospital^c^	6 (25)	2 (33)	0
	Other^c^	2 (8)	0	0

Data are n (%)

Abbreviations: NHS = National Health Service; TSC = tuberous sclerosis complex

^a^ Only caregivers who reported that their partner/spouse is involved in the care for the individual with TSC.

^b^ Only caregivers who reported that their parent, sibling, child, or other relative is involved in the care for the individual with TSC.

^c^ These responses were only for participants who answered “Yes” to use of NHS services.

Over half of caregivers (56%) reported receiving no financial support. Half of the caregivers (50%) utilized NHS counseling, while 29% had accessed respite care. Use of NHS services by other household members is also provided in Table 1.

### Impact of caring on the Caregiver’s Work Productivity and Career

The full range of comments from caregivers on the impact of TSC on their work productivity and career are detailed in eTable 1 in the Additional file 1. Some caregivers (n = 4) have had to stop working completely, while others (n = 6) have had to reduce their working hours or change job to ensure more flexible hours:

[It is] not possible to work when having multiple seizures during the night. [I] have recently given up my job.

I was only ever able to work part time to fit around caring needs. However, as time has gone on, and I have got older, I have needed to reduce my working hours.

In addition, several caregivers described missing out on promotions or having to change to more junior positions at work (n = 7):

I have never been able to apply for promotion because my time and energy are limited.

One caregiver stated that they had no choice but to work as they did not receive a disability allowance (n = 1). Some caregivers reported they had understanding employers (n = 3) who allowed them to work flexible hours, while others described the physical and psychological toll of having to work while caring (n = 4):

It has been physically and emotionally draining to work and care at the same time, and my physical and mental health have been severely impacted.

### Experience of Shared decision-making in relation to ASM

**Fig. 2:**
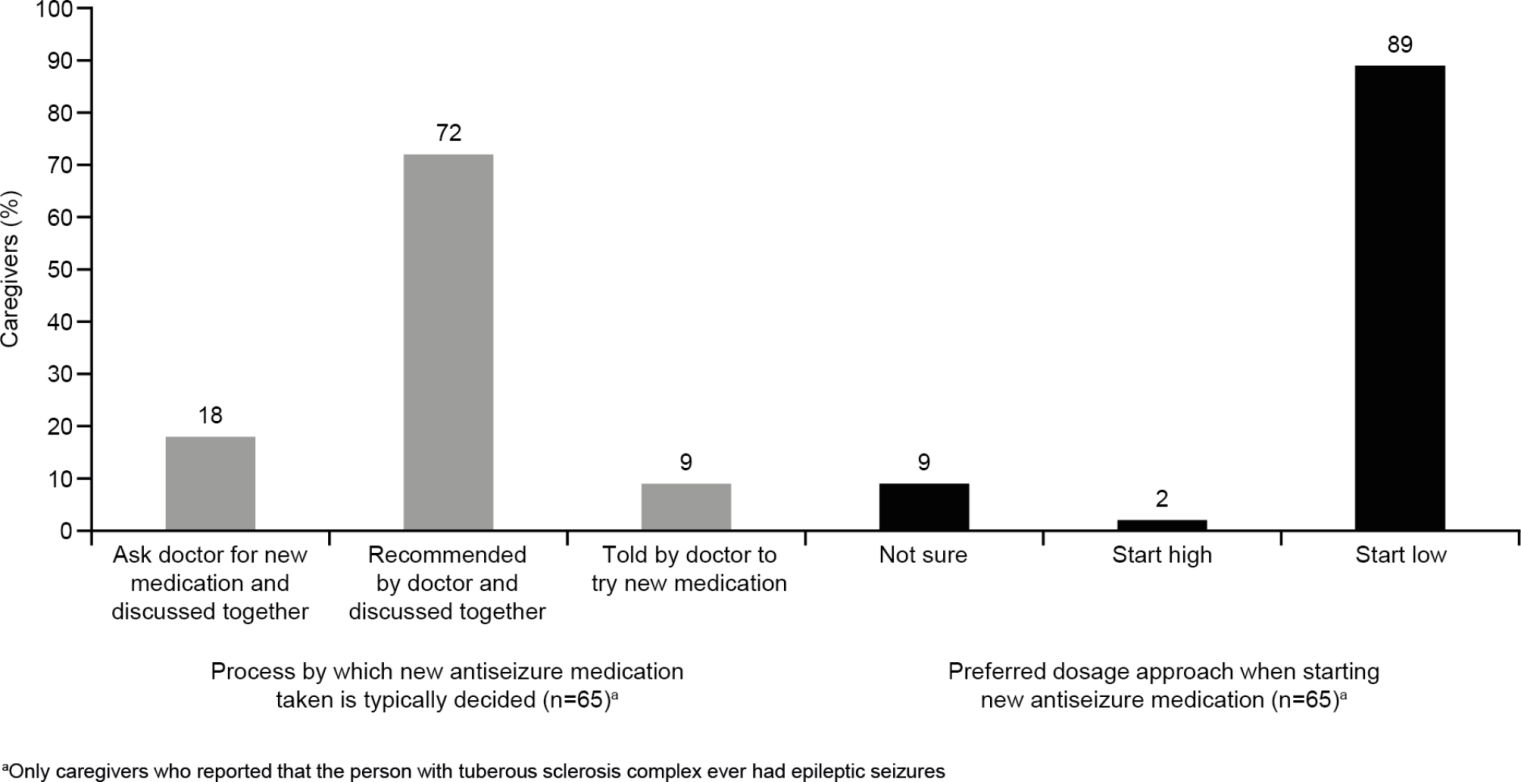
Experience of shared decision-making in relation to the ASM taken by the individual with TSC Abbreviations: ASM = antiseizure medication; TSC = tuberous sclerosis complex

Caregivers’ experiences of shared decision-making when starting a new ASM are summarized in Fig. 2. Most (72%) caregivers reported receiving recommendations about new treatments from their doctor and then discussing the treatment together, and a high proportion of caregivers (89%) preferred that treatment was initiated at a low dose.

### Caregiver satisfaction with TSC Healthcare Services used by the individual they **care for**

While most caregivers (69%) were satisfied or extremely satisfied with pediatric TSC healthcare services, only 25% were satisfied or extremely satisfied with the transition to adult TSC healthcare services (Table 2).

**Table 2:** Caregiver satisfaction with TSC healthcare services used by the individual they care for

	Extremely unsatisfied/unsatisfied, n (%)	Neither unsatisfied nor satisfied, n (%)	Extremely satisfied/satisfied, n (%)
**Satisfaction with pediatric healthcare services (n = 71)**	12 (17)	10 (14)	49 (69)
**Satisfaction with transition from pediatric to adult healthcare services (n = 40)** ^**a**^	19 (48)	11 (28)	10 (25)
**Satisfaction with adult healthcare services (n = 38)** ^**b**^	9 (24)	11 (29)	18 (47)

Abbreviation: TSC, tuberous sclerosis complex

^a^Only caregivers who reported that the individual with TSC was 16 or older.

^b^Only caregivers who reported that the individual with TSC was 18 or older.

The full range of free-text comments from caregivers on the transition from pediatric to adult healthcare services for TSC are detailed in eTable 2 in the Additional file 1. One caregiver reported that they could not recall a transition period:

Is there supposed to be a transition!! Not that we participated in. [The] next appointment was in an adult clinic, and that was that.

The percentage of caregivers that reported being satisfied or extremely satisfied in adult TSC healthcare services (47%) was lower than that for pediatric care. Several caregivers reported that the adult healthcare services felt more disjointed compared with pediatric services (n = 7):

[…] As a child, all services, neurology, kidneys, behavior, were all in one hospital, and [a] consultant seemed to have an all over care of him. As an adult, everything [is] very disjointed, different hospitals for different things, [they] don’t seem to look at him as a whole person where everything is interconnected.

Other caregivers perceived a lack of support from social services (n = 3) or thought that healthcare professionals in adult services lacked knowledge about TSC and were not supportive (n = 5):

It is a living nightmare. People [healthcare professionals] don’t listen, don’t understand, and if it wasn’t for legal deputyship, he’d probably not still be alive now.

### Impact of the COVID-19 pandemic on individuals with TSC and caregivers

**Fig. 3:**
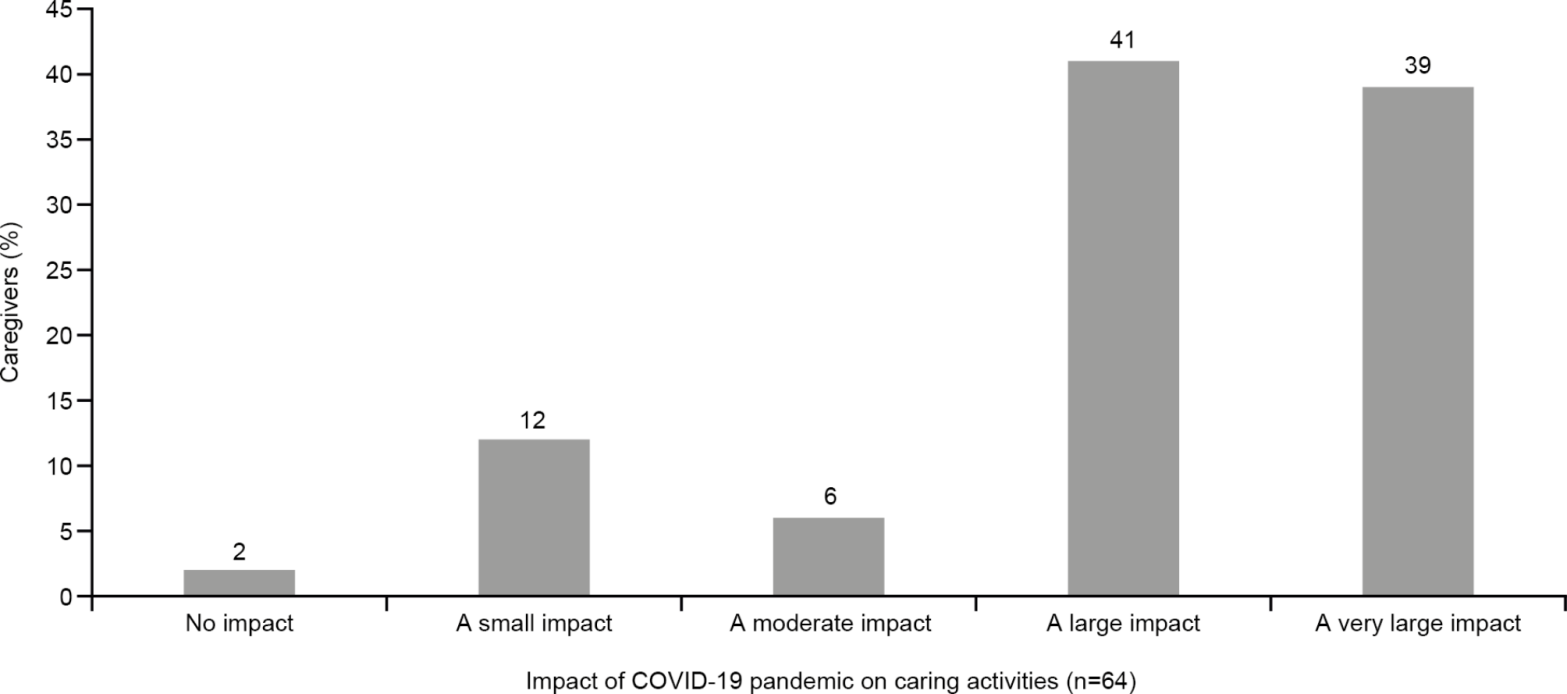
Impact of COVID-19 pandemic on the caregiver Abbreviation: COVID-19 = coronavirus disease 2019

In total, 80% of caregivers indicated that the COVID-19 pandemic had a “large” or “very large” impact on their caring activities, while only 2% of participants stated that COVID-19 had no impact (Fig. 3). For all free-text comments on the impact of COVID-19, see eTable 3 in the Additional file 1.

Many caregivers (n = 21) reported feeling overwhelmed and isolated:

My daughter was shielding throughout the pandemic. This meant that we were shut in mostly, support stopped, and family didn’t visit. It has been stressful; I am suffering with a degree of undiagnosed depression in my opinion.

It’s been tough, felt isolated, overwhelmed but worse for [name of child].

The pandemic also affected caregivers’ ability to work:

My daughter was shielding due to taking immune suppressant[s], and it was unsafe for me to work due to high risk.

Further to the impact on the caregiver, the pandemic had an impact on the emotional wellbeing and behavior of individuals with TSC, with caregivers reporting worsening behavior difficulties (n = 2), distress and anxiety (n = 5), and frustration (n = 3) due to changes in routine and the need to shield:

The whole household has been shielding, which was extremely difficult as she didn’t understand what was going on nor about social distancing.

The pandemic has also caused my daughter to suffer more from anxiety, stress, and maybe depression.

It has made home life very difficult as frustrations run high. There is nothing open or available to help my child’s development.

Moreover, due to the need to shield and the closure of leisure venues, caregivers (n = 6) reported struggling to keep individuals with TSC entertained.

Many caregivers (n = 12) additionally reported difficulties with booking medical appointments and accessing services during the COVID-19 pandemic (eTable 3 in the Additional file 1):

Reduced therapy, paperwork required for further support not done, entry into specialist school delayed, MRI [magnetic resonance imaging] and scans delayed, eye checks delayed, kidney checks delayed, development profiles delayed.

## Discussion

Overall, this survey provides novel and valuable insights into the caregiver experience of looking after individuals with TSC. Our results show that caregivers largely feel involved in decisions concerning new seizure treatments for the individual with TSC, and the majority are satisfied with pediatric healthcare services for children with TSC. In contrast, many caregivers highlighted the need for an improved transition from pediatric to adult healthcare services, and fewer were satisfied with adult healthcare services for TSC compared with pediatric care. The survey also showed that COVID-19 has considerably affected caregivers and individuals with TSC.

This survey reports data on caregivers’ experience of shared decision-making and the caregivers’ preference for starting new medication with a low dose. This “low and slow” approach with ASM is one that is widely accepted by clinicians and is recommended clinically in most situations [11]. This approach may help mitigate the risk of experiencing adverse events, thus supporting measures to aid adherence [11].

While most caregivers were satisfied with TSC healthcare services, there appears to be a particular need for an improved transition from pediatric to adult care. Addressing concerns and needs of caregivers during this transition period has been highlighted previously for individuals with TSC [12, 13]. In a multicenter survey study of 60 adults with TSC and epilepsy in France, one-third of participants indicated they had not undergone a transition process, and of those that did, 25% did not experience “continuity” between services, and 40% did not rate their experience as “good” [13]. In another study, 16 young adults (aged 17–30 years) with TSC and 12 parents of individuals with TSC who had transitioned into adult care across the Netherlands were interviewed. Several problems were identified during the transition process by this study, including the following: loss of familiar connection with the pediatrician; lack of a coordinating physician with an overview of the patient’s history; absence of consultation between physicians; less personalized care for adult patients; no available process for parents to discuss concerns with physicians; and no easy access to a contact person [12].

Similar experiences in the transition from pediatric to adult healthcare services in other chronic conditions have also been reported, with parents desiring greater involvement in the transition process and some feeling their role in the process was not recognized [14]. Effective, standardized transition programs are important to support clinical outcomes, [14] yet little evidence exists on the effectiveness of interventions designed to improve the transition from pediatric to adult health services in chronic childhood conditions [15]. While different models of transition do exist that can be applied in TSC, [16] there remains a need for an established model for the transition from pediatric to adult care in TSC that health services can adopt and follow.

The current survey also showed that the impact of the COVID-19 pandemic on TSC caregiver burden was considerable, particularly relating to difficulties associated with shielding and the inability to access medical appointments. To our knowledge, no other literature exists on the impact of COVID-19 on the burden of care for caregivers of individuals with TSC. We expect that further insights will come to light on this topic and how this impact has evolved throughout the pandemic. We have observed that these experiences during the COVID-19 pandemic are similar to those of parent caregivers to individuals with disabilities more generally [17]. For example, in a survey of 4074 participants from the UK who cared for children with disabilities, the majority (up to 80%) reported worsening emotional and mental health for their children or themselves [17]. As a result of the lockdowns during the pandemic, some parents reported that they had not sought necessary medical help for their children with disabilities (44%), themselves or their partner (54%), or their children without disabilities (17%). Additionally, families reported financial pressures with either a reduction in income (39%) or increased costs (61%) [17].

While this study provides novel data and insights, it should be noted that there are some limitations to consider, including the small sample size, the high participant drop-out rate, the potential for non-representativeness as recruitment used convenience sampling via an open invitation, and reliance on self-reporting by caregivers. Future studies on this topic should aim to mitigate these limitations by using larger sample sizes and suitable methods/study designs.

## Conclusions

In summary, this study provides important information about the experiences of caregivers of individuals with TSC. Many caregivers would engage in discussions together with their dependent’s doctor when discussing seizure treatments and preferred starting with a low dose. Caregivers highlighted a need for improvement in the transition of care from pediatric to adult services. Furthermore, the COVID-19 pandemic has considerably affected individuals with TSC and their caregivers by impacting caring activities and leaving many feeling overwhelmed and isolated.

## Electronic supplementary material

Below is the link to the electronic supplementary material.

Supplementary Material 1

## Data Availability

The sponsor is adhering to current US and EU requirements so will not make individual deidentified participant data available; all relevant data are provided with the manuscript and supporting files.
