# Supplementary material for: Shared decision-making and the caregiver experience in tuberous sclerosis complex: results from a UK survey
Source: Orphanet J Rare Dis. 2023 Apr 11;18:78. doi: 10.1186/s13023-023-02677-7 (PMC10088777; doi:10.1186/s13023-023-02677-7)
Supplement: Supplementary file 1 — Supplementary Material 1 [file 13023_2023_2677_MOESM1_ESM.docx]

Additional file 1

eTable 1. Comments about the impact of caring on the caregiver’s work and career

| **Response category** | **Example quotes** |
| --- | --- |
| **Impacts on career** | |
| Some caregivers had to stop working completely (n = 4) | *“[It is] not possible to work when having multiple seizures during the night. [I] have recently given up my job.”* |
| Others had to reduce their working hours or change to have a more flexible job (n = 6) | *“I was only ever able to work part time to fit around caring needs. However, as time has gone on, and I have got older, I have needed to reduce my working hours.”* |
| Several caregivers described missing out on promotions or having to change to more junior positions at work (n = 7) | *“I have never been able to apply for promotion because my time and energy are limited.”*  *“I’ve had to change career and take a lesser paid job to fit everything in.”* |
| One caregiver stated that they had no choice but to work as they did not receive a disability allowance (n = 1) | *“I can’t not work because we don’t receive disability [allowance], but it is becoming increasingly difficult, and I have a lot of time off.”* |
| **Ability to maintain job** | |
| A few reported still being able to work while the individual is at school (n = 2) | *“I work during the school day, and in [the]  holidays, I have her with me; my bosses allow me to do that.”* |
| Some caregivers reported still being able to work due to having an understanding employer, who allows them to work flexible hours, or to take the individual into the workplace when not at school (n = 3) | *“I’ve been lucky enough to work for understanding employers part time and very much appreciate this privilege.”*  *“In [the] holidays, I have her with me; my bosses allow me to do that. If she has a seizure at school, I can pick her up and return to work.”* |
| **Consequences with regards to HRQL** | |
| Some caregivers described finding it physically and emotionally draining to maintain a job, as well as be a full-time caregiver (n = 4) | *“Our daughter has many hospital appointments, and we have meetings with agencies in order to seek support as her behavior is challenging, and so I had to reduce my hours, and then over a year ago, I had to resign because of stress. I haven’t slept properly for the past 27 years, and this has impacted on my health.”*  *“It has been physically and emotionally draining to work and care at the same time, and my physical and mental health have been severely impacted.”* |
| One caregiver, who no longer worked, mentioned that they missed the support of and friendship with their colleagues (n = 1) | *“I miss having colleagues for support and friendship. I have only been a full-time carer for the last 7 months.”* |

Abbreviation: HRQL = health-related quality of life.

eTable 2. Comments about the individual’s transition from pediatric to adult healthcare services

| **Response category** | **Example quotes** |
| --- | --- |
| **Negative responses** | |
| Adult healthcare services were perceived to be more disjointed compared with pediatric services, where different specialists were all in the same building. This resulted in caregivers struggling to coordinate the individual’s numerous appointments and access certain healthcare specialists (n = 7) | *“Care felt [d]isjointed as pediatric services are under one roof, [whereas] adults have to see a variety of different specialists.”*  *“We have ended up accessing dermatology, for instance, via a GP referral when things became unmanageable. We have never seen a renal doctor in adult healthcare. It is always via the neurologist who asks for an opinion from his colleague if I push for one.”*  *“[…] As a child, all services, neurology, kidneys, behavior, were all in one hospital, and [a] consultant seemed to have an all over care of him. As an adult, everything [is] very disjointed, different hospitals for different things, [they] don’t seem to look at him as a whole person where everything is interconnected.”* |
| One caregiver perceived that there was no transition period and that the individual had their first appointment at adult healthcare services straight after their last appointment at pediatric services (n = 1) | *“Is there supposed to be a transition!! Not that we participated in. [The] next appointment was in an adult clinic, and that was that.”* |
| Healthcare professionals in adult services were perceived to lack knowledge about TSC and not be supportive toward the caregiver’s and individual’s needs (n = 5) | *“[It] was difficult due to lack of professionals with knowledge of TSC.”*  *“It is a living nightmare. People [healthcare professionals] don’t listen, don’t understand, and if it wasn’t for legal deputyship, he’d probably not still be alive now.”* |
| There was also a perceived lack of support from social services, once the individual had reached adulthood (n = 3) | *“[It] was a difficult transition from pediatric support in a residential school to care in the community, with fairly adequate services some 20 years ago, but now [it is] becoming more and more difficult due to social services cuts and placements now private [and] not under county council remit.”* |
| **Positive responses** | |
| One caregiver felt that the transition period was well organized and that there was time for joint clinics with both pediatric and adult healthcare professionals before the individual was discharged from pediatric services (n = 1) | *“It was well planned, and time was allowed for joint clinics (adult and pediatric together). She was not discharged from pediatrics until we were satisfied with the transition.”* |
| Two caregivers of adults with TSC reported that the individual still attended a TSC clinic, where they benefitted from receiving support from a multidisciplinary team (n = 2) | *“Transition to adult neurology was fine. Fortunately, our daughter accesses a TSC clinic which monitors her other symptoms.”* |

Abbreviations: GP = general practitioner; TSC = tuberous sclerosis complex.

**eTable 3.** Comments about the impact of the COVID-19 pandemic

| **Response category** | **Example quotes** |
| --- | --- |
| **Impact on the individual with TSC’s daily activities** | |
| Many caregivers mentioned difficulty with obtaining medical appointments and access to therapy sessions due to the closure and canceling of these services (n = 12) | *“It has made it more difficult to have regular appointments with those involved in my child’s care.”*  *“Reduced therapy, paperwork required for further support not done, entry into specialist school delayed, MRI and scans delayed, eye checks delayed, kidney checks delayed, development profiles delayed.”* |
| Several caregivers reported struggling to keep the individual entertained due to the closure of leisure venues (e.g., swimming pools) and the need for the family to shield (n = 6) | *“Leisure activities (e.g., horse riding for disabled, swimming, etc.) also stopped. The whole household has been shielding, which was extremely difficult as she didn’t understand what was going on nor about social distancing.”* |
| **Impact on the individual with TSC’s emotional wellbeing and behavior** | |
| Two caregivers described how the individual’s behavior difficulties had worsened due to the change to their daily routine (n = 2) | *“Day centers [are] closed. My son was shielding, so I did not use any carers within [the] house for a long period. His weight increased, his sleep pattern is out of sorts, [and] his behavior has deteriorated.”* |
| Several caregivers mentioned that the  individual became distressed and anxious  due to not understanding the change to their daily life (n = 5) | *“The whole household has been shielding, which was extremely difficult as she didn’t understand what was going on nor about social distancing.”*  *“The pandemic has also caused my daughter to suffer more from anxiety, stress, and maybe depression.”* |
| Others reported that the individual became frustrated because of not being able to leave the house due to their need to shield (n = 3) | *“[It] had a huge impact – [I] couldn’t go out at all, frustration, and regression.”*  *“It has made home life very difficult as frustrations run high. There is nothing open or available to help my child’s development.”* |
| **Impact on caregiver** | |
| Many caregivers reported feeling overwhelmed and isolated because of their lack of social support and respite due to their family’s need to shield (n = 21) | *“My daughter was shielding throughout the pandemic. This meant that we were shut in mostly, support stopped, and family didn’t visit. It has been stressful; I am suffering with a degree of undiagnosed depression in my opinion.”*  *“It’s been tough, felt isolated, overwhelmed but worse for [name of child].”* |
| A few caregivers reported feeling anxious  about the possibility of the individual getting  the virus (n = 2) | *“It’s been hard from [a] mental health point of view shielding him [and] worrying about him getting the virus.”*  *“Stress of keeping him safe.”* |
| Several caregivers reported that their work was impacted due to needing to stay at home to shield and home school their child (n = 6) | *“My daughter was shielding due to taking immune suppressant[s], and it was unsafe for me to work due to high risk.”*  *“For several months, I was working from home and providing 24 hour a day care because having someone else in my home would have been too intrusive.”* |

Abbreviations: COVID-19 = coronavirus 2019; MRI = magnetic resonance imaging;
TSC = tuberous sclerosis complex.
